# Stroke burden and associated predictors in Ghana: a Bayesian analysis of the WHO study on global ageing and adult health

**DOI:** 10.1007/s40520-026-03462-9

**Published:** 2026-07-20

**Authors:** Abdul-Karim Iddrisu, Adam Mohammed

**Affiliations:** 1 Department of Statistics, Demography and Population Studies, Faculty of Natural and Applied Sciences, University of Botswana, Gaborone, Botswana; 2 Department of Mathematics and Statistics, University of Energy of Natural Resources, Sunyani, Ghana

**Keywords:** Adult, Aged, Diabetes mellitus, Ghana, hypertension, Obesity, Prevalence, Risk factors, Stroke

## Abstract

**Background:**

Stroke is a major public health concern in sub-Saharan Africa, including Ghana. Although its clinical and demographic risk factors are well established globally, nationally representative evidence on stroke prevalence and associated factors in Ghana remains limited. Also, previous research using WHO SAGE Wave 1 data has examined stroke prevalence and associated factors among older adults in Ghana; however, important methodological gaps remain, particularly regarding the explicit consideration of the complex survey design and unobserved cluster-level heterogeneity.

**Aim:**

This study examined the prevalence and risk factors of stroke among adults in Ghana.

**Methods:**

Data were obtained from the World Health Organization’s Study on Global AGEing and Adult Health (WHO SAGE, Wave 1), comprising 5,066 Ghanaian participants, including individuals aged 50 years and older and a comparative group aged 18–49 years. Descriptive statistics and chi-square tests were used to assess variable distributions and bivariate associations with stroke. Given the hierarchical and complex survey structure of the data, with individuals nested within primary sampling units (PSUs), survey multivariable logistic regression was applied to estimate adjusted associations, while Bayesian multilevel logistic regression models were fitted to account for cluster-level random variation and improve estimate stability. Analyses were conducted in R (v4.5.1) using the survey and R-INLA packages, with statistical significance set at *p* < 0.05.

**Results:**

The two models produced consistent findings, with the Bayesian multilevel model offering the most stable and precise estimates after accounting for cluster-level heterogeneity at the PSU level and the within-cluster dependence among sampled individuals. Cluster-level heterogeneity was negligible (ICC = 0.0001; 95% CrI: 0.0000–0.0005), indicating minimal residual variation in stroke across PSUs. The Bayesian multilevel logistic regression analysis identified several significant predictors of stroke. Individuals who had ever attended school had higher odds of stroke compared to those who had not (aOR = 1.48, 95% CrI: 1.00-2.21). Obesity (aOR = 1.68, 95% CrI: 1.05–2.70), arthritis (aOR = 1.74, 95% CrI: 1.08–2.79), diabetes (aOR = 2.67, 95% CrI: 1.53–4.66), and hypertension (aOR = 3.46, 95% CrI: 2.28–5.23) were all associated with increased odds of stroke. Participants aged 50 years and above also had higher odds compared to younger adults (aOR = 2.76, 95% CrI: 1.18–6.44), whereas engagement in vigorous work-related physical activity was associated with reduced odds of stroke (aOR = 0.60, 95% CrI: 0.38–0.95).

**Conclusion:**

Public health strategies should emphasize physical activity, healthy diets, weight control, and improved management of chronic diseases. These efforts align with the sustainable development goal 3 (SDG 3); Good Health and Well-being, particularly Target 3.4, which aims to reduce premature mortality from non-communicable diseases through prevention and treatment by 2030.

## Introduction

The Global Burden of Disease (GBD) 2021 estimates identify stroke as the third leading cause of mortality worldwide, accounting for approximately 7.3 million deaths (10.7% of all deaths), surpassed only by ischaemic heart disease and COVID-19 [1]. Stroke also ranked as the fourth leading cause of disability-adjusted life-years (DALYs), contributing 160.5 million DALYs, or 5.6% of the global disease burden. Feigin et al. [1, 2] reported a marked increase in the global stroke burden from 1990 to 2021, largely attributable to modifiable risk factors. In 2021, the estimated global prevalence of stroke was 93.8 million cases, with 11.9 million new cases occurring in the same year [1]. Low- and lower-middle-income countries (LMICs) shoulder the greatest share, accounting for 87.2% of worldwide stroke deaths and 89.4% of stroke-related DALY [1, 2]. The financial implications are equally striking, with the global cost of stroke exceeding US$890 billion annually equivalent to 0.66% of global GDP and expected to almost double by 2050 [2, 3].

In Africa, stroke poses a particularly pressing challenge. Incidence rates reach as high as 316 per 100,000 population, with especially elevated prevalence in West Africa [4]. While stroke was once considered uncommon on the continent less than a century ago [5], it has now emerged as a leading driver of DALYs across all African subregions, comparable to conditions such as migraine and meningitis [4, 6]. Current data further suggest that Africa carries some of the highest indices of stroke burden globally [7, 8].

In Africa, the growing stroke burden is driven by multiple life-course determinants. Early-life undernutrition increases cardiometabolic vulnerability in adulthood [4], a risk compounded by air pollution exposure [4], population aging, and dietary transitions [9, 10]. Furthermore, for every clinically recognized stroke, an estimated four silent strokes occur typically undiagnosed yet strongly linked to long-term cognitive decline and mental health complications [9, 10].

Over the past decade, stroke research in Africa has expanded considerably, yielding deeper insights into its aetiologies and risk factors, including associations with sickle cell disease, HIV/AIDS, and COVID-19 [4]. Current investigations also extend to stroke outcomes, rehabilitation, economic costs, quality of life, and complications such as cognitive decline, dementia, and seizures [8]. Advances in genetics, including genome-wide association studies (GWAS), are beginning to elucidate hereditary contributors to stroke risk in African populations [4, 11]. Nevertheless, substantial gaps persist in research, clinical care, and health policy. Akinyemi et al. [4, 8] provide a comprehensive review of the African stroke landscape, underscoring distinctive epidemiological and genetic features while outlining priorities for more effective prevention and management.

In Ghana, stroke incidence has been rising steadily and is now the first among the top three causes of mortality [12, 13]. Reported cases more than doubled between 2016 and 2021, increasing from 10,732 to 23,009, a 61% rise [12, 13]. The condition is particularly common among individuals with hypertension and diabetes [21, 23] and accounts for the leading cause of death among females (56.5%) and the fifth among males (43.5%). Evidence from a multicenter cohort study across five hospitals further confirmed the heightened risk of stroke among patients with hypertension and/or type 2 diabetes [12, 14]. Despite being largely preventable, awareness of stroke risk factors and early warning signs remains limited, especially in Accra [14].

Researchers have consistently emphasized the lack of reliable data on stroke incidence, prevalence, mortality, and disability in Africa, compounded by the absence of established surveillance systems at both national, regional levels and individual level [4]. This challenge is particularly pronounced in Sub-Saharan Africa, including Ghana, where stroke statistics remain scarce [15, 16]. Despite the growing stroke burden in Ghana, the application of advanced modeling approaches to forecast stroke trends, identify stroke predictors, at both regional and individual level, remains limited [3]. For instance, in Ghana, several studies have investigated the determinants and outcomes of stroke, but these have primarily employed conventional frequentist approaches. For example, Sarfo and colleagues [14] conducted a multicenter prospective cohort study of adults with hypertension and type 2 diabetes, following participants across five public hospitals to evaluate risk of incident stroke. Using multivariate Cox-proportional hazards models, they identified smoking and physical inactivity as important predictors of stroke incidence. A related study [17] from the same cohort assessed baseline kidney function and showed that reduced estimated glomerular filtration rate (eGFR) predicted increased stroke risk, again using Cox regression methods.

Other hospital-based studies have applied logistic regression techniques to evaluate functional outcomes and mortality among stroke patients. For instance, a study at Korle Bu Teaching Hospital compared outcomes between stroke patients with and without diabetes and found that comorbid diabetes was associated with worse survival and recovery [18]. Similarly, findings from the large multicenter Stroke Investigative Research and Educational Network (SIREN) highlighted determinants of stroke mortality across West Africa, including Ghana, using adjusted odds ratios from logistic regression models [19]. At the population level, analyses of routine data from the Ghana Health Service documented a 61% rise in reported stroke cases between 2016 and 2021. These studies employed negative binomial regression and basic geospatial mapping to examine temporal trends and geographic disparities [20].

However, the analytical approaches used in previous studies of individual-level stroke data in Ghana often present important methodological limitations [3, 17, 19]. In particular, many rely on classical regression techniques, assume that observations are independent, and fail to adequately account for the hierarchical nature of the data, where patients may be nested within households, communities, primary sampling units (PSU) districts, or regions. Failure to account for such clustering may lead to underestimated standard errors and potentially misleading inferences. While previous research [21] has assessed stroke prevalence and associated factors among older adults in Ghana using WHO SAGE Wave 1 data, there is limited evidence from studies that explicitly account for the complex survey design and unobserved cluster-level heterogeneity. Therefore, the present study extends existing literature by applying both survey-weighted [22, 23] and Bayesian multilevel [24] logistic regression approaches to provide more robust evidence on individual-level factors associated with stroke in Ghana. This contributes to the limited empirical evidence on stroke in Sub-Saharan Africa, Ghana and offering insights that can underpin future epidemiological research and health policy development.

## Design and methods

### Study design

**Fig. 1:**
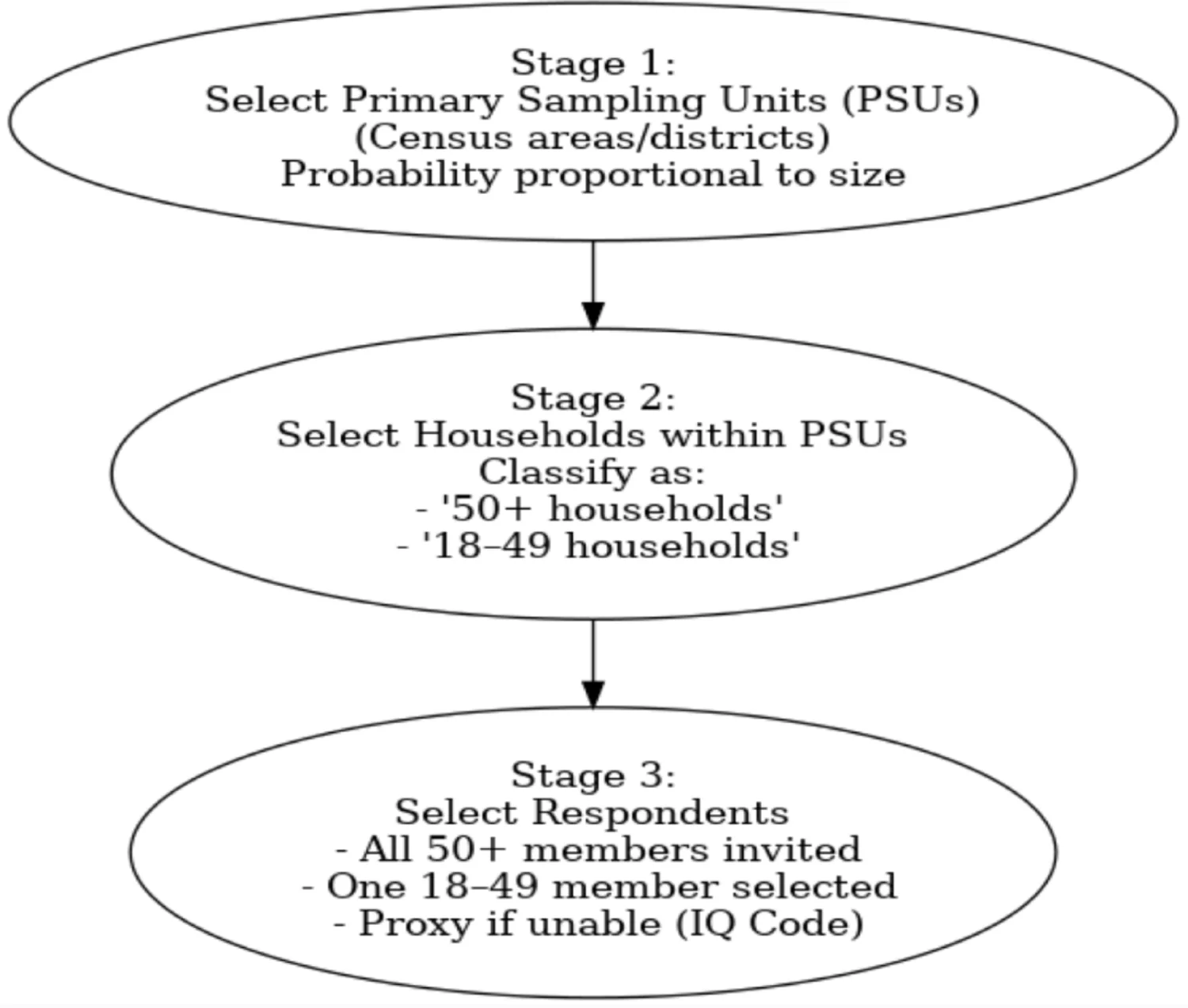
Flow-chart of the study design

This study analysed secondary data from the World Health Organization’s Study on Global AGEing and Adult Health (WHO SAGE Waves 1) using the Ghana sample. Details of the original survey design, sampling procedures, and data collection methods have been published elsewhere [25, 26]. Information on study design and sample selection procedure are presented in Figure 1 in **Appendix****A**.

### Study variables

#### Outcome variable

The outcome variable in this study is a binary stroke status (takes values of 1 if participant has ever been told that he/she has stroke and 0 if no).

#### Predictor variables

Previous studies have identified a range of factors associated with stroke risk [1, 27–31]. In this study, the potential predictors of stroke considered include socio-demographic characteristics and health-related risk factors. Socio-demographic variables comprise age (coded as 1 for ≥ 50 years and 0 for 18–49 years), Gender (1 = male, 0 = female), educational attainment (1 = ever attended school, 0 = never attended school), and place of residence (1 = rural, 0 = urban). Health and lifestyle risk factors considered include body mass index (BMI: 3 = obese [≥ 30.0], 2 = overweight [25.0–29.9], 1 = underweight [< 18.5], 0 = normal [18.5–24.9]); tobacco use (1 = ever smoked, 0 = never smoked); alcohol consumption (1 = ever consumed alcohol, 0 = never consumed alcohol); physical activity (1 = work involves vigorous exercise, 0 = does not involve vigorous exercise); arthritis (1 = ever diagnosed, 0 = never diagnosed); angina (1 = ever diagnosed, 0 = never diagnosed); diabetes mellitus (DM) (1 = ever diagnosed, 0 = never diagnosed); chronic lung disease (CLD) (1 = ever diagnosed, 0 = never diagnosed); asthma (1 = ever diagnosed, 0 = never diagnosed); and hypertension (1 = ever diagnosed, 0 = never diagnosed), and perceived social trust was included (1 = has someone to trust, 0 = has no one to trust).

### Statistical analysis

The statistical analysis was conducted using both design-based and Bayesian survey modelling approaches to examine the association between stroke and its potential risk factors. Descriptive statistics were used to summarize participant characteristics and chi-square tests of association was applied to assess bivariate associations between stroke and the explanatory variables [32, 33].

#### Survey logistic regression

At the multivariable level, a survey logistic regression model was first fitted to estimate unadjusted and adjusted odds ratios (unOR and aOR respectively) and corresponding 95% confidence intervals (CIs). This model served as the primary design-based analytical approach and allowed for valid population-level inferences by accounting for unequal probabilities of clustering [22]. The survey logistic regression model was fitted using the *survey package* in R, with Primary Sampling Units (PSUs) specified as the clustering variable. This design-based approach accounted for the complex sampling structure of the data by adjusting for the correlation of observations within the same PSU.

#### Bayesian multilevel modelling

To complement the primary analysis (survey regression), a Bayesian multilevel logistic regression model [34, 35], which accounts for the hierarchical structure of the data with PSUs as clustering variable was employed. This approach allows us to capture unobserved cluster-level heterogeneity at the PSU level, while simultaneously estimating the association between individual-level covariates and stroke. By modelling PSUs as random effect, the model adjusts for intra-cluster correlation and provides more accurate estimates of standard errors and credible intervals. This hierarchical specification aligns with the survey design, ensuring that cluster-level variations are properly accounted for in the analysis. Through the incorporation of prior distributions for model parameters, the Bayesian approach provided an alternative and potentially more stable estimation framework, particularly in the context of sparse or rare outcomes and small subgroup sizes [36]. Posterior estimates were presented as unadjusted and adjusted odds ratios with 95% credible intervals (CrIs).

#### Model fitting and parameter estimation

We first fitted a survey logistic regression model using the *survey package* in R to obtain weighted estimates of the relationships between stroke, a binary outcome, and various covariates. Building on this, we then applied a Bayesian multilevel logistic regression model using the Integrated Nested Laplace Approximation (INLA) package in R [37], modelling stroke as a Bernoulli-distributed outcome with a logit link function. The model included factors such as education, location, BMI, arthritis, angina, diabetes, chronic lung disease, asthma, hypertension, age, trust, and exercise, with PSUs to capture hierarchical clustering. To ensure reproducibility, we specified weakly informative Gaussian priors (mean = 0, variance = 1000) for the fixed effects, while the random intercept for PSUs were assigned independent Gaussian distributions with log-gamma hyperpriors on the precision parameters (shape = 1, rate = 0.01). Finally, we calculated the intraclass correlation coefficient (ICC) to estimate how much of the variation in stroke risk could be attributed to differences between clusters at the PSU level. We also performed model diagnostics using density plots for the posterior estimates from Bayesian multilevel model and discrimination was assessed using the concordance statistic (area under the receiver operating characteristic curve, AUC) based on predicted probabilities from the survey logistic regression model. To ensure that the predictors included in the logistic regression model were not highly correlated, we assessed multicollinearity using the Variance Inflation Factor (VIF).

## Results

We first present the descriptive statistics of the variables used in this study, followed by the results of the chi-square test of association. Subsequently, we report findings from the univariable survey logistic regression and Bayesian multilevel logistic models, and finally, the multivariable survey logistic regression and Bayesian multilevel logistic model.

### Descriptive statistics of variables used in the study

Table 1 presents the descriptive characteristics of the 5,066 study participants. The overall prevalence of stroke in the sample was 2.37%. Of the 5,066 respondents included in the analysis, 53.28% were male and 46.72% were female, showing a slight predominance of male participants in the sample. Nearly half of the respondents (49.45%) had ever attended school, and slightly more than half (55.29%) were residents of urban areas. Regarding body mass index, the majority of participants (55.61%) were of normal weight, while 18.52% were overweight, 11.78% were obese, and 14.09% were underweight. In relation to lifestyle factors, 24.34% reported ever smoking tobacco, whereas 58.61% had ever consumed alcohol. With respect to self-reported health conditions, 11.41% had arthritis, 3.47% had diabetes, 12.12% had hypertension, 3.40% had asthma, 2.92% had angina, and 0.55% had chronic lung disease. Most respondents (84.23%) were aged 50 years and above. Furthermore, 77.32% reported having someone they could trust, and 46.19% indicated that their work involved vigorous physical activity.

**Table 1:** Percentage distribution of study variables (*N* = 5,057)

Variable	Category	*n* (%)
Ever had stroke?	No	4,946 (97.63)
	Yes	120 (2.37)
Gender		
	Female	2,367 (46.72)
	Male	2,699 (53.28)
Ever attended school?	No formal education	2,561 (50.55)
	Ever attended school	2,505 (49.45)
Geographical location	Rural	2,265 (44.71)
	Urban	2,801 (55.29)
BMI	Underweight	714 (14.09)
	Normal weight	2,817 (55.61)
	Overweight	938 (18.52)
	Obese	597 (11.78)
Ever smoke tobacco?	No	3,833 (75.66)
	Yes	1,233 (24.34)
Ever consumed alcohol?	No	2,097 (41.39)
	Yes	2,969 (58.61)
Ever diagnosed with arthritis?	No	4,488 (88.59)
	Yes	578 (11.41)
Ever diagnosed with angina?	No	4,918 (97.08)
	Yes	148 (2.92)
Ever diagnosed with diabetes?	No	4,890 (96.53)
	Yes	176 (3.47)
Chronic Lung Disease (CLD)?	No	5,038 (99.45)
	Yes	28 (0.55)
Ever diagnosed with asthma?	No	4,894 (96.60)
	Yes	172 (3.40)
Ever diagnosed with hypertension?	No	4,452 (87.88)
	Yes	614 (12.12)
Age category	18–49 years	799 (15.77)
	≥ 50 years	4,267 (84.23)
Have someone to trust?	No	1,149 (22.68)
	Yes	3,917 (77.32)
Work involves vigorous exercise?	No	2,726 (53.81)
	Yes	2,340 (46.19)

### Chi-square test of association between stroke status and individual level characteristics

Table 2 summarizes the bivariate associations between stroke status and selected sociodemographic, behavioural, psychosocial, and clinical characteristics. The chi-square test revealed statistically significant associations between stroke and educational attainment, geographical location, body mass index (BMI), arthritis, angina, diabetes, chronic lung disease (CLD), asthma, hypertension, age category, social trust, and vigorous work activity.

Stroke prevalence was significantly higher among respondents who had ever attended school (3.0%) compared with those who had never attended school (1.8%) (χ² = 8.377, *p* = 0.004). A significant urban–rural disparity was also observed, with urban residents reporting a higher prevalence of stroke (3.2%) than rural residents (1.3%) (χ² = 19.316, *p* < 0.001). A significant relationship was found between BMI and stroke status (χ² = 43.073, *p* < 0.001). Stroke prevalence increased progressively across BMI categories, from 1.0% among underweight individuals to 1.9% among those with normal BMI, 2.5% among overweight individuals, and 6.0% among obese individuals, suggesting a dose-response pattern between excess body weight and stroke occurrence.

Several pre-existing health conditions were significantly associated with stroke. Participants with arthritis had more than double the prevalence of stroke compared with those without arthritis (4.5% vs. 2.1%; χ² = 12.794, *p* < 0.001). Similarly, stroke prevalence was higher among respondents with angina (5.4% vs. 2.3%; χ² = 6.079, *p* = 0.014), diabetes (10.8% vs. 2.1%; χ² = 55.986, *p* < 0.001), CLD (10.7% vs. 2.3%; χ² = 8.480, *p* = 0.004), and asthma (6.4% vs. 2.2%; χ² = 12.483, *p* < 0.001). Among all variables examined, hypertension demonstrated the strongest association with stroke. Respondents with hypertension had a stroke prevalence of 9.0%, compared with 1.5% among those without hypertension (χ² = 131.160, *p* < 0.001). Stroke was also significantly more common among older adults aged ≥ 50 years (2.7%) than among those aged 18–49 years (0.8%) (χ² = 10.736, *p* = 0.001), underscoring the role of ageing as an important correlate of stroke.

Psychosocial and occupational characteristics were likewise associated with stroke status. Respondents who reported having no one to trust had a higher prevalence of stroke (3.2%) compared with those who reported having someone to trust (2.1%) (χ² = 4.659, *p* = 0.031). In addition, participants whose work did not involve vigorous physical activity had a substantially higher prevalence of stroke (3.3%) than those engaged in vigorous work (1.2%) (χ² = 23.986, *p* < 0.001). In contrast, no statistically significant associations were observed between stroke status and gender (χ² = 0.295, *p* = 0.587), tobacco smoking (χ² = 0.002, *p* = 0.965) or alcohol consumption (χ² = 0.016, *p* = 0.900).

**Table 2:** Distribution of stroke status by sociodemographic, lifestyle, and health-related characteristics with chi-square tests of association

Variable	Category	Stroke: 0, *n* (%)	Stroke: 1, *n* (%)	χ²	*p*-value
**Gender**	Female	2,308 (97.51)	59 (2.49)	0.295	0.587
	Male	2,638 (97.74)	61 (2.26)		
Ever attended school?					
	No	2,516 (98.24)	45 (1.76)	8.377	0.004
	Yes	2,430 (97.01)	75 (2.99)		
Geographical location					
	Rural	2,235 (98.68)	30 (1.32)	19.316	< 0.001
	Urban	2,711 (96.79)	90 (3.21)		
BMI					
	Underweight	707 (99.02)	7 (0.98)	43.073	< 0.001
	Normal	2,763 (98.08)	54 (1.92)		
	Overweight	915 (97.55)	23 (2.45)		
	Obese	561 (93.97)	36 (6.03)		
Ever smoked tobacco?					
	No	3,742 (97.63)	91 (2.37)	0.002	0.965
	Yes	1,204 (97.65)	29 (2.35)		
Ever consumed alcohol?					
	No	2,048 (97.66)	49 (2.34)	0.016	0.900
	Yes	2,898 (97.61)	71 (2.39)		
Ever diagnosed with arthritis?					
	No	4,394 (97.91)	94 (2.09)	12.794	< 0.001
	Yes	552 (95.50)	26 (4.50)		
Ever diagnosed with angina?					
	No	4,806 (97.72)	112 (2.28)	6.079	0.014
	Yes	140 (94.59)	8 (5.41)		
Ever diagnosed with diabetes?					
	No	4,789 (97.93)	101 (2.07)	55.986	< 0.001
	Yes	157 (89.20)	19 (10.80)		
Ever diagnosed with CLD?					
	No	4,921 (97.68)	117 (2.32)	8.480	0.004
	Yes	25 (89.29)	3 (10.71)		
Ever diagnosed with asthma?					
	No	4,785 (97.77)	109 (2.23)	12.483	< 0.001
	Yes	161 (93.60)	11 (6.40)		
Ever diagnosed with hypertension					
	No	4,387 (98.54)	65 (1.46)	131.160	< 0.001
	Yes	559 (91.04)	55 (8.96)		
Age category					
	18–49	793 (99.25)	6 (0.75)	10.736	< 0.001
	≥ 50	4,153 (97.33)	114 (2.67)		
Have someone to trust?					
	No	1,112 (96.78)	37 (3.22)	4.659	0.031
	Yes	3,834 (97.88)	83 (2.12)		
Work involves vigorous exercise?					
	No	2,635 (96.66)	91 (3.34)	23.986	< 0.001
	Yes	2,311 (98.76)	29 (1.24)		

### Results from univariable survey logistic regression and Bayesian multilevel logistic regression models

We carried out two sets of univariable analyses to explore how different factors relate to stroke risk: a traditional survey logistic regression and a Bayesian multilevel logistic regression. The results from both approaches are summarized in Table 3.

**Table 3:** Unadjusted odds ratios (unaOR) with corresponding standard error (SE) and 95% confidence interval (95%CI) from univariable survey logistic regression and 95% credible intervals (95%CrI) from the Bayesian multilevel logistic regression model

Variable	Category	Survey logistic regression			Bayesian multilevel logistic regression		
		unaOR	SE	95%CI *P*-values	unOR	SE	95CrI
**Gender**							
	Female (Ref)	-	-	-	-	-	-
	Male	0.90	0.194	(0.62, 1.32) 0.606	0.90	0.185	(0.63, 1.30)
**Ever been to school?**							
	No (Ref)	-	-	-	-	-	-
	Yes	1.73	0.199	(1.17, 2.55) 0.007	1.73	0.191	(1.19, 2.52)
**Geographical location**							
	Rural (Ref)	-	-	-	-	-	-
	Urban	2.47	0.233	(1.67, 3.65) <0.001	2.50	0.213	(1.65, 3.80)
**BMI**							
	Normal weight (Ref)	-	-	-	-	-	-
	Underweight	0.51	0.410	(0.23, 1.13) 0.098	0.48	0.404	(0.22, 1.05)
	Overweight	1.29	0.281	(0.74, 2.23) 0.040	2.27	0.252	(0.78, 2.08)
	Obese	3.28	0.22	(2.12, 5.09) < 0.001	3.27	0.22	(2.12, 5.04)
**Ever smoked tobacco?**							
	No (Ref)	-	-	-	-	-	-
	Yes	0.99	0.214	(0.65, 1.51) 0.964	0.98	0.216	(0.64, 1.50)
**Ever drink alcohol?**							
	No (Ref)	-	-	-	-	-	-
	Yes	1.02	0.194	(0.70, 1.50) 0.903	1.03	0.188	(0.71, 1.48)
**Ever been diagnosed with arthritis?**							
	No (Ref)	-	-	-	-	-	-
	Yes	2.20	0.239	(1.38, 3.52) 0.001	2.17	0.226	(1.39, 3.39)
**Ever been diagnosed with angina?**							
	No (Ref)	-	-	-	-	-	-
	Yes	2.45	0.364	(1.20, 5.00) 0.015	2.32	0.376	(1.11, 4.85)
**Ever been diagnosed with diabetes?**							
	No (Ref)	-	-	-	-	-	-
	Yes	5.74	0.277	(3.33, 9.88) <0.001	5.63	0.263	(3.36, 9.43)
**Ever been diagnosed with chronic lung disease?**							
	No (Ref)	-	-	-	-	-	-
	Yes	5.05	0.602	(1.55, 16.43) 0.008	4.40	0.618	(1.31, 14.77)
**Ever been diagnosed with asthma?**							
	No (Ref)	-	-	-	-	-	-
	Yes	3.00	0.361	(1.48, 6.08) 0.003	2.89	0.326	(1.52, 5.48)
**Ever been diagnosed with hypertension?**							
	No (Ref)	-	-	-	-	-	-
	Yes	6.64	0.181	(4.66, 9.47) <0.001	6.64	0.189	(4.58, 9.61)
**Age category**							
	18–49 (Ref)	-	-	-	-	-	-
	≥ 50	3.63	0.418	(1.60, 8.23) 0.002	3.92	0.421	(1.72, 8.95)
**Have anyone you can trust?**							
	No (Ref)	-	-	-	-	-	-
	Yes	0.65	0.211	(0.43, 0.98) 0.043	0.66	0.201	(0.44, 0.97)
**Does your work involve vigorous exercise?**							
	No (Ref)	-	-	-	-	-	-
	Yes	0.36	0.243	(0.23, 0.58) <0.001	0.36	0.215	(0.24, 0.55)

Overall, the results from the two models were very similar. The estimated odds ratios and their intervals were largely consistent, with the direction and strength of associations agreeing across models. This agreement suggests that the observed associations are reliable and not heavily influenced by the choice of statistical method. When comparing precision, the standard errors and interval widths were generally comparable. However, for factors with relatively few cases like chronic lung disease or angina, the Bayesian model produced slightly more conservative estimates with narrower credible intervals. This demonstrates the Bayesian approach’s strength in stabilizing estimates when data are sparse or rare. Additionally, the Bayesian credible intervals provide a direct probabilistic interpretation, indicating the likelihood that the true odds ratio falls within the interval, which can be more intuitive in public health applications. For these reasons, it is the preferred model for subsequent analyses, particularly when exploring multivariable relationships. Significant factors identified here will form the basis for subsequent multivariable modelling.

### Results from multivariable survey logistic and Bayesian multilevel logistic regression models

Table 4 presents the result from the multivariable survey logistic and Bayesian multilevel logistic regression models. The survey logistic regression and Bayesian multilevel logistic regression models showed largely consistent results, with both models identifying hypertension, diabetes, obesity, arthritis, and older age as key factors associated with stroke. In contrast, geographical location, angina, chronic lung disease, and trust/social support did not demonstrate clear independent associations with stroke after controlling for other covariates.

**Fig. 2:**
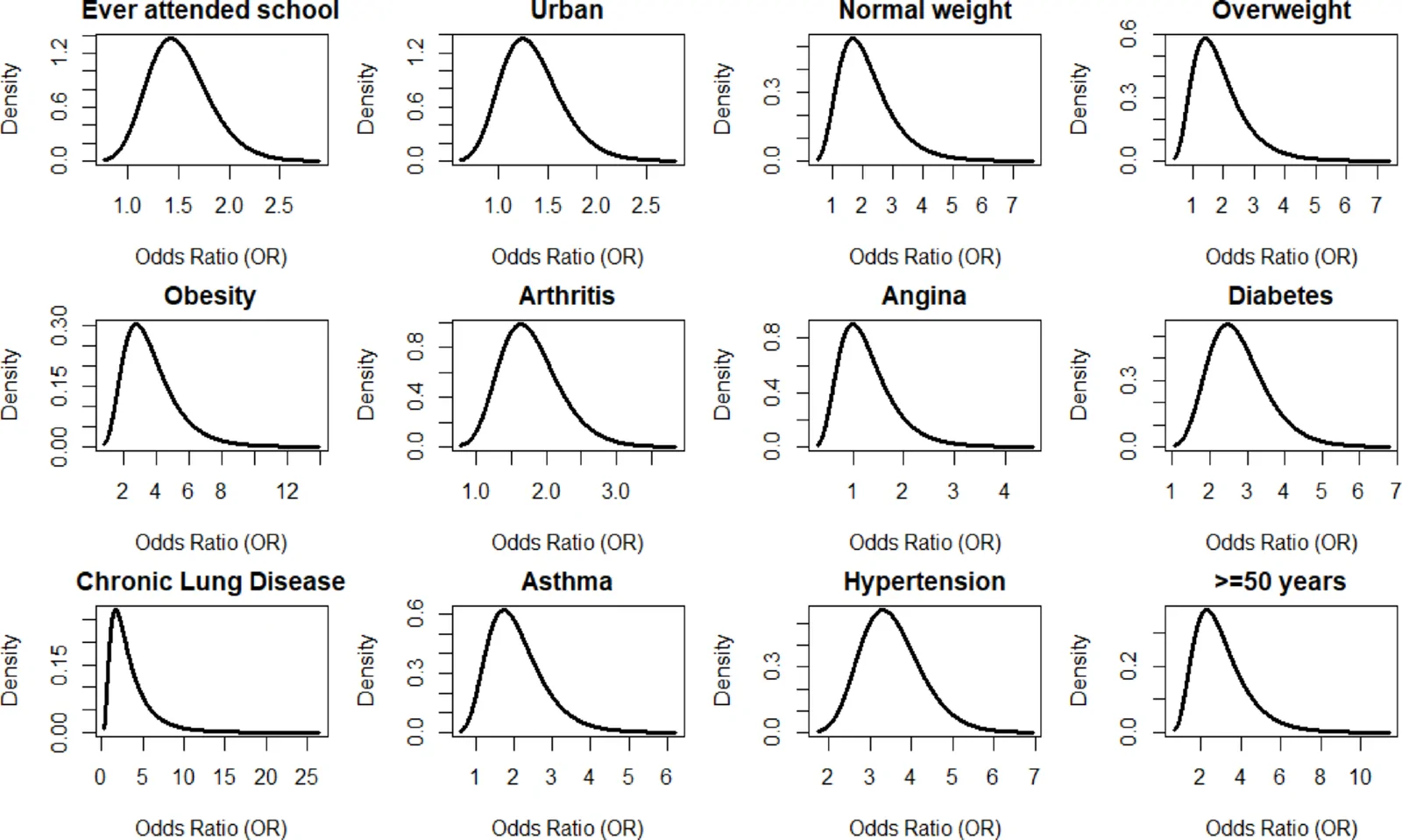
Bayesian multilevel model: parameters convergence diagnostics plots

**Fig. 3:**
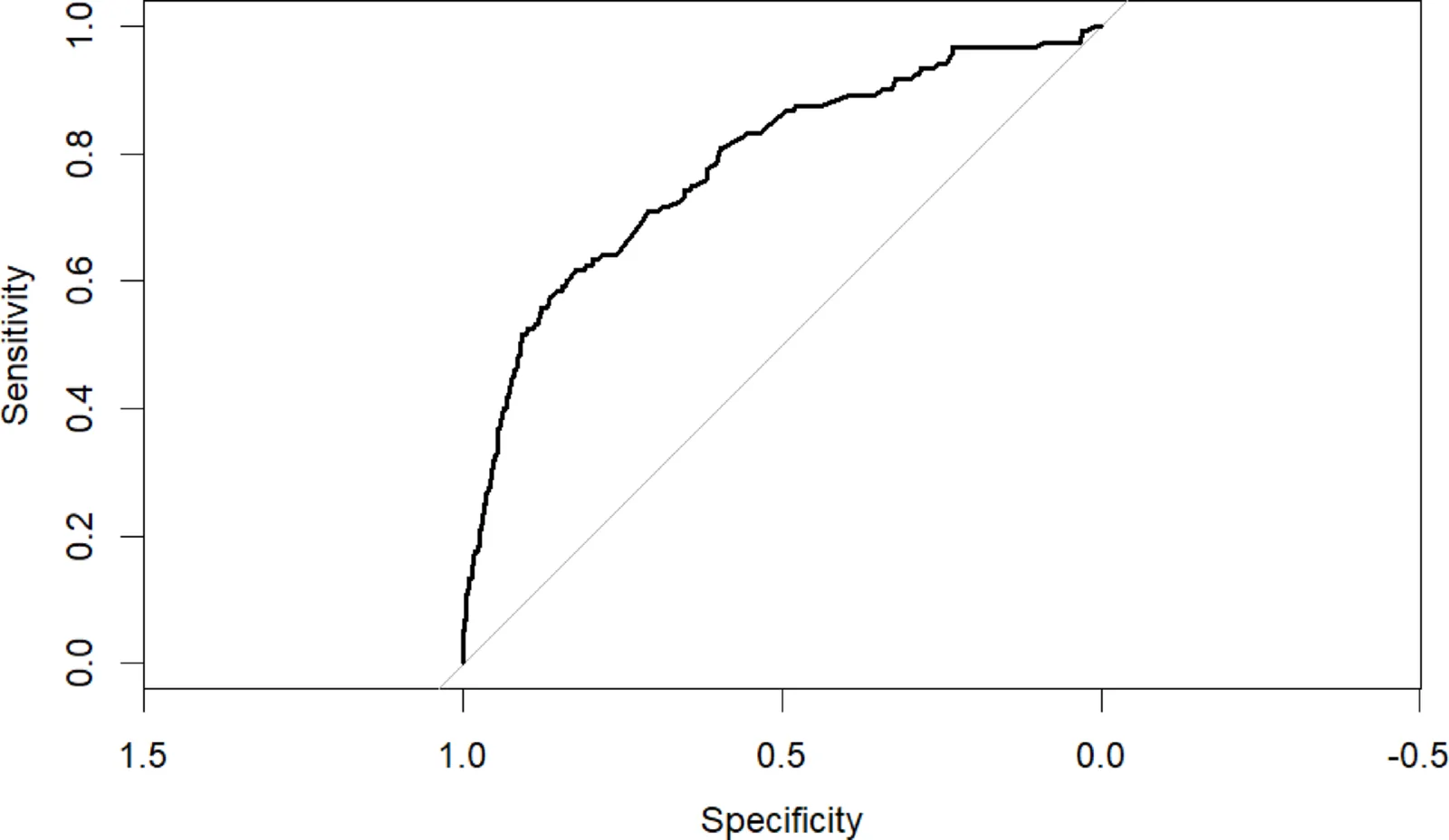
Survey logistic regression model: Receiver Operating Characteristic (ROC) curve

Despite the general consistency between the two approaches, some differences were observed. The Bayesian multilevel model yielded slightly more stable and precise estimates for education, vigorous exercise, and asthma. For instance, while vigorous exercise appeared to be protective in both models, the evidence for this association was stronger in the Bayesian analysis. Given that the Bayesian multilevel model explicitly accounted for PSU-level clustering and offered more robust estimation in the presence of a relatively rare stroke outcome, it was considered the more appropriate model for reporting the primary results. Figure 2**in** **Appendix****B** illustrates that the parameter estimates from the Bayesian multilevel model have converged. Also, the PSU-level random intercept variance was close to zero (posterior mean = 0.0002; 95% CrI: 0.0000–0.0017), indicating negligible unobserved heterogeneity across clusters. Similarly, the intraclass correlation coefficient (ICC) was extremely small (0.0001; 95% CrI: 0.0000–0.0005), suggesting that only about 0.01% of the unexplained variation in stroke was attributable to differences between primary sampling units (PSUs). These findings indicate that residual variation in stroke was largely explained at the individual level rather than the cluster level. For the survey logistic regression model, the area under the receiver operating characteristic curve (AUC) implies that the model has a reasonable ability (approximately 80%) to correctly distinguish between individuals with and without stroke (Figure 3 in **Appendix****C)**. To ensure that the predictors included in the survey logistic regression model were not highly correlated, we assessed multicollinearity using the Variance Inflation Factor (VIF). All variables had values below 1.2, indicating minimal multicollinearity. This suggests that the regression coefficients are stable and can be interpreted reliably.

**Table 4:** Adjusted odds ratios (aOR) with corresponding standard error (SE) and 95% confidence/credible intervals (95%CI/CrI) from multivariable survey logistic regression, Bayesian and multilevel logistic regression model

Variable	Category	Survey logistic regression			Bayesian multilevel logistic regression		
		aOR	SE	95% CI *P*-value	aOR	SE	95% CrI
**Ever been to school?**							
	No (Ref)	-	-	-	-	-	-
	Yes	1.48	0.213	(0.97, 2.24) 0.069	1.48	0.202	(1.00, 2.21)
**Geographical location**							
	Rural (Ref)	-	-	-	-	-	-
	Urban	1.30	0.258	(0.78, 2.15) 0.315	1.31	0.231	(0.83, 2.06)
**BMI**							
	Normal weight (Ref)	-	-	-	-	-	-
	Underweight	0.54	0.416	(0.24, 1.21) 0.137	0.50	0.408	(0.23, 1.12)
	Overweight	0.87	0.290	(0.50, 1.53) 0.637	0.86	0.262	(0.51, 1.44)
	Obese	1.68	0.245	(1.04, 2.72) 0.035	1.68	0.242	(1.05, 2.70)
**Ever been diagnosed with arthritis?**							
	No (Ref)	-	-	-	-	-	-
	Yes	1.75	0.266	(1.04, 2.95) 0.036	1.74	0.241	(1.08, 2.79)
**Ever been diagnosed with angina?**							
	No (Ref)	-	-	-	-	-	-
	Yes	1.23	0.439	(0.52, 2.91) 0.364	1.18	0.409	(0.53, 2.63)
**Ever been diagnosed with diabetes?**							
	No (Ref)	-	-	-	-	-	-
	Yes	2.69	0.283	(1.54, 4.68) < 0.001	2.67	0.283	(1.53, 4.66)
**Ever been diagnosed with chronic lung disease?**							
	No (Ref)	-	-	-	-	-	-
	Yes	3.01	0.698	(0.77, 11.81) 0.116	2.71	0.693	(0.70, 10.54)
**Ever been diagnosed with asthma?**							
	No (Ref)	-	-	-	-	-	-
	Yes	2.01	0.386	(0.94, 4.28) 0.072	1.96	0.350	(0.99, 3.90)
**Ever been diagnosed with hypertension?**							
	No (Ref)	-	-	-	-	-	-
	Yes	3.43	0.220	(2.23, 5.27) < 0.001	3.46	0.212	(2.28, 5.23)
**Age category**							
	18–49 (Ref)	-	-	-	-	-	-
	≥ 50	2.56	0.429	(1.11, 5.95) 0.030	2.76	0.432	(1.18, 6.44)
**Have anyone you can trust?**							
	No (Ref)	-	-	-	-	-	-
	Yes	0.69	0.226	(0.44, 1.08) 0.104	0.69	0.208	(0.46, 1.05)
**Does your work involve vigorous exercise?**							
	No (Ref)	-	-	-	-	-	-
	Yes	0.61	0.266	(0.36, 1.02) 0.060	0.60	0.232	(0.38, 0.95)
PSU-level variance (95%CrI)		-	-	-	0.0002 (0.0000-0.0017)		
ICC (95%CrI)		-	-	-	0.0001 (0.0000–0.0005)		

The Bayesian multilevel logistic regression results showed that individuals who had ever attended school had higher odds of stroke compared to those who had not (aOR = 1.48, 95% CrI: 1.00-2.21) and those diagnosed with obesity was significantly associated with higher odds of stroke compared to those with normal (aOR = 1.68, 95% CrI: 1.05–2.70). Similarly, respondents who had ever been diagnosed with arthritis had significantly increased odds of stroke compared to those without the condition (aOR = 1.74, 95% CrI: 1.08–2.79). Among the chronic conditions examined, diabetes and hypertension were also significantly associated with stroke. Individuals diagnosed with diabetes had 2.67 times higher odds of stroke compared to those without diabetes (aOR = 2.67, 95% CrI: 1.53–4.66), while those with hypertension had 3.46 times higher odds (aOR = 3.46, 95% CrI: 2.28–5.23). Age was another important predictor, as respondents aged 50 years and above had significantly higher odds of stroke compared to those aged 18–49 years (aOR = 2.76, 95% CrI: 1.18–6.44). In contrast, respondents whose work involved vigorous physical activity had significantly lower odds of stroke compared to those whose work did not involve vigorous exercise (aOR = 0.60, 95% CrI: 0.38–0.95), suggesting a protective effect.

## Discussion

This study identified several sociodemographic, behavioural, and health-related factors significantly associated with the outcome under investigation. The findings highlight that education, obesity, chronic health conditions (arthritis, diabetes, and hypertension), and older age increased the likelihood of the outcome, whereas vigorous physical activity appeared to confer a protective effect. These findings are largely consistent with previous research, though some patterns highlight important contextual nuances.

Individuals who had ever attended school were more likely to experience stroke compared to those who had never attended school. This association may reflect better health awareness, access to diagnosis, and health-seeking behaviours among the educated population. Similar trends have been reported in recent studies across sub-Saharan Africa and other low- and middle-income countries, where higher educational attainment often corresponds with greater access to healthcare services and increased likelihood of disease detection [38, 39]. However, contrasting evidence from high-income settings suggests that higher education is typically protective, possibly due to greater health literacy and healthier lifestyle practices [40]. This divergence highlights the role of contextual socioeconomic and health system differences in shaping risk patterns.

Body mass index was another key determinant, with obesity showing a strong positive association with the outcome. The observed relationship aligns with prior evidence linking obesity to metabolic and cardiovascular complications that elevate chronic disease risk [41, 42]. Excess body fat contributes to systemic inflammation, insulin resistance, and endothelial dysfunction, which collectively heighten vulnerability to multiple adverse health outcomes. Similar findings in Ghana and other African populations underscore the growing public health challenge of obesity-related morbidity amid rapid urbanization and changing dietary patterns [43–45].

Consistent with existing literature, individuals diagnosed with arthritis, diabetes, hypertension, and asthma had significantly higher odds of the stroke. These results are in line with prior epidemiological studies demonstrating that chronic comorbidities often cluster and exacerbate overall disease risk [46, 47]. The strong associations with diabetes and hypertension, in particular, agree with prior studies linking these chronic conditions to a heightened risk of multimorbidity and poor health outcomes [48, 49]. The comorbidity burden of noncommunicable diseases (NCDs) is increasingly recognized in sub-Saharan Africa, driven by epidemiological transitions and aging populations [50]. Similarly, the positive association between arthritis and the outcome is in line with findings from recent studies showing that inflammatory musculoskeletal conditions often coexist with other chronic diseases and contribute to physical limitations and reduced quality of life [51, 52]. The observed association between asthma and the outcome also supports evidence that chronic respiratory conditions are linked with systemic inflammation and elevated risk for other comorbid conditions [52].

Age emerged as an important determinant, with adults aged 50 years and above exhibiting more than twice the odds of the outcome compared to younger adults. This finding reflects the well-documented pattern of increased risk of chronic health conditions with advancing age [53, 54]. Age-related physiological decline, cumulative exposure to risk factors, and reduced resilience to disease likely contribute to this pattern. The finding emphasizes the need for age-targeted preventive interventions and health screening programs aimed at older populations.

Conversely, engagement in vigorous physical activity was associated with reduced odds of the outcome, indicating its potential protective role. This finding is supported by numerous studies demonstrating the benefits of regular physical activity in reducing cardiometabolic and inflammatory risks [55, 56]. Physical activity has been identified as a key modifiable factor that can mitigate the adverse effects of obesity, hypertension, and diabetes, particularly in resource-constrained settings [57]. Vigorous physical activity improves cardiovascular fitness, modulates immune response, and reduces obesity-related risks, thereby mitigating the likelihood of adverse health outcomes. The protective effect observed here underscores the importance of promoting physical activity as part of population-level health interventions.

### Conclusion

This study provides robust evidence that sociodemographic, behavioral, and health-related factors jointly influence the likelihood of experiencing stroke. The findings highlight that education, obesity, chronic health conditions (arthritis, diabetes, hypertension, and asthma), and older age significantly increase the risk, while vigorous physical activity reduces it. These results are largely consistent with existing literature but also reveal important contextual differences that reflect the unique epidemiological and socioeconomic environment of the study population.

The positive association between education and stroke may reflect greater health awareness and access to diagnosis among the educated, rather than an intrinsic increase in risk. Similarly, the strong associations with obesity and chronic conditions reaffirm the growing burden of noncommunicable diseases in sub-Saharan Africa, driven by urbanization, changing lifestyles, and population aging. The protective role of vigorous physical activity further highlights the importance of modifiable lifestyle factors in mitigating chronic disease risk. Overall, the study underscores the multifactorial nature of stroke and related health outcomes, emphasizing the interplay between demographic, behavioral, and health system factors in shaping population health patterns.

### Recommendations

Health authorities should prioritize integrated NCD prevention and management programs that target obesity, hypertension, diabetes, and other chronic conditions through early screening, continuous monitoring, and community-based interventions. Public health campaigns should emphasize the importance of regular vigorous physical activity and balanced diets to reduce the risk of stroke and related conditions. Workplace wellness programs, community exercise initiatives, and urban planning that promotes active transport can help achieve this goal.

Given the heightened vulnerability among older adults, routine health screening and age-targeted health promotion interventions should be expanded to facilitate early detection and management of chronic diseases in this group. Educational programs should not only focus on increasing health literacy but also emphasize the adoption of preventive health behaviors across all educational levels. This can help bridge the knowledge–practice gap observed between educational attainment and health outcomes. Addressing the growing burden of stroke and other chronic diseases requires collaboration between the health sector, education, agriculture, urban development, and social welfare sectors. Policies should adder.

ss the upstream social and environmental determinants of health that contribute to obesity, sedentary lifestyles, and poor dietary habits.

Investments in diagnostic infrastructure, workforce training, and primary care strengthening are crucial to improve early diagnosis and management of chronic diseases, particularly in underserved regions. Further longitudinal and mechanistic studies are recommended to clarify the causal pathways linking education, lifestyle behaviors, and chronic comorbidities to stroke risk, as well as to assess the effectiveness of targeted interventions in reducing disease burden across different population subgroups.

### Limitations

This study has several limitations that warrant consideration. The stroke outcome was based on self-reported data from the WHO Study on Global AGEing and Adult Health (SAGE), which may be affected by recall bias and misclassification, particularly in settings with limited diagnostic confirmation. As a result, underreporting or overreporting of stroke events is possible, potentially biasing prevalence estimates. Also, self-reported variables like physical activity may be influenced by social desirability bias. Although the WHO SAGE dataset is comprehensive, its reliance on proxy responses introduces possible information errors. Cultural and language differences may also affect response accuracy. The focus on adults aged 50 years and above limits generalizability to younger populations.

## Data Availability

The dataset analyzed in the current study are secondary data and publicly available from the World Health Organization’s Study on Global AGEing and Adult Health (SAGE) via the WHO Multi-Country Studies Data Archive: [SAGE Waves](https:/www.who.int/data/data-collection-tools/study-on-global-ageing-and-adult-health/sage-waves) . Access to the data requires registration and approval of a brief online request. The specific dataset used was WHO SAGE Ghana Waves 1 and 2.

## Appendix A: Study design and population

A WHO SAGE Waves 1 and 2 employed a multistage cluster sampling design to ensure nationally representative samples. In this design, sampling was carried out in sequential stages. First, primary sampling units (PSUs), typically census enumeration areas or districts, were selected using probability proportional to size methods. Within each selected PSU, households were randomly chosen and classified into two categories: “50+ households” (with at least one member aged 50 years or older), which is the focus of this study and comparative sample of those aged “18–49 households.” Finally, within these households, individual respondents were selected according to the study’s age-based eligibility criteria. For participants unable to respond independently, proxy respondents were identified based on cognitive assessment using the IQ Code [58]. This approach allowed SAGE to achieve broad population coverage while maintaining feasibility and cost-effectiveness, particularly in low- and middle-income country settings where national household listings may be limited. The use of clustering also enabled efficient fieldwork logistics while ensuring that both older adults (primary focus) and younger adults (comparative group) were adequately represented. Figure 1 presents the flow chart of the study design.

## Appendix C: Model diagnostics: survey logistic regression model

The survey logistic regression model demonstrated an area under the curve (AUC) of 0.779, indicating acceptable discrimination. This means that the model has a reasonable ability to correctly distinguish between individuals with and without stroke. In practical terms, there is approximately a 77.9% probability that the model will assign a higher predicted probability of stroke to a randomly selected individual who actually had a stroke compared to a randomly selected individual who did not. This level of discriminatory performance suggests that the included covariates provide meaningful predictive information for identifying stroke risk in the study population.
